# Coexistent sickle cell anemia and autoimmune hemolytic anemia in two adolescents

**DOI:** 10.31744/einstein_journal/2024RC1105

**Published:** 2024-11-06

**Authors:** Vinícius Reis Soares, Bruna Paccola Blanco, Carla Luana Dinardo, Marlene Pereira Garanito

**Affiliations:** 1 Universidade de São Paulo Hospital das Clínicas Faculdade de Medicina São Paulo SP Brazil Hospital das Clínicas, Faculdade de Medicina, Universidade de São Paulo, São Paulo, SP, Brazil.; 2 Universidade de São Paulo Hospital das Clínicas Faculdade de Medicina São Paulo SP Brazil Fundação Pró-Sangue, Hospital das Clínicas, Faculdade de Medicina, Universidade de São Paulo, São Paulo, SP, Brazil.

**Keywords:** Anemia,hemolytic,autoimmune, Adolescent, Anemia,sickle cell, Autoantibodies, Autoimmunity

## Abstract

The development of alloantibodies or autoantibodies is a complication observed in sickle cell disease. Autoimmunization occurs in 7.6-12% of chronically or intermittently transfused patients with sickle cell disease; however, the clinical implications of autoAbs are unclear. Few studies have focused on pediatric sickle cell disease and autoimmune hemolytic anemia. Herein, we present the coexistence of sickle cell disease and autoimmune hemolytic anemia in two adolescent patients, focusing on their pathophysiology, diagnosis, clinical management, and outcomes.

## INTRODUCTION

Alloimmunization and autoimmunization to red blood cell (RBC) antigens is a complication that occurs in sickle cell disease (SCD). The development of RBC autoantibodies (autoAbs) occurs in 7.6-12% of chronically or intermittently transfused patients with SCD and is associated with a 57-92% incidence of previous alloimmunization.^(1-3)^

Although the clinical implications of autoAbs in patients with SCD remain unclear, they may be associated with severe and potentially life-threatening autoimmune hemolytic anemia (AIHA).^(1,2,4)^

To the best of our knowledge, we report the first Brazilian pediatric cases of coexisting sickle cell anemia and AIHA.

## CASE REPORTS

### Case 1

Case 1 was a 12-year-old male patient with A+ blood type, no parental consanguinity, and no personal or family medical history of AIHA, diagnosed with Hashimoto's thyroiditis, HbSS disease, and had splenectomy, hip vascular necrosis, chronic pain, and was under treatment with hydroxyurea (HU), and chronic transfusion therapy. He first received blood transfusion at 10 months of age. Before the start of chronic transfusion therapy (at 11 years of age), the patient received 24 sporadic transfusions of RBC units. He received 73 RBC units matched for ABO, RH (D, C, c, E, e), and Kell (K) antigens without developing alloantibodies (alloAbs).

The patient was admitted to our hospital with mucocutaneous pallor, jaundice (worsening from baseline), mild tachycardia (heart rate of 106 bpm), normal respiratory rate (20 breaths per minute), and oxygen saturation (98%). His general condition was good. Cardiac auscultation revealed a systolic murmur, but the liver was not palpable.

**Figure 1:**
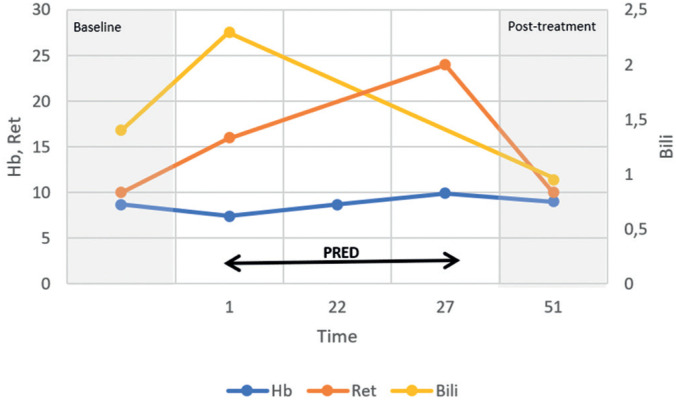
Progression of laboratory markers and clinical interventions during autoimmune hemolytic anemia in sickle cell disease (Case 1)

Blood tests showed worsening of anemia [hemoglobin (Hb) from 8.7g/dL (baseline) to 7.4g/dL], an increased reticulocyte absolute number [from 142 × 10^9^/L (baseline) to 430 × 10^9^/L], and an increased indirect bilirubin level [from 1.4mg/dL (baseline) to 2.3mg/dL]. White blood cell (WBC) and platelet counts were normal. A direct antiglobulin test (DAT) revealed an undetermined IgG (panagglutinin) and the presence of the complement fraction C3d. No alloAbs were detected. These data, along with the clinical and laboratory signs of hemolysis, led us to confirm the diagnosis of warm AIHA (wAIHA). Infection, primary immunodeficiency disorder, lymphoproliferative diseases, and solid malignancies were excluded. The patient was treated with oral prednisone (2.0mg/kg/day with a maximum dose of 60mg/day for 28 days, followed by weaning until complete withdrawal after 8 weeks). The patient was managed without RBC transfusion. One month after the start of treatment, the patient's Hb level, reticulocyte count, and indirect bilirubin level were at their baseline values, and after 6 months, the DAT result became negative. One year later, the DAT result was positive owing to a warm autoantibody (IgG), with no signs of increased hemolysis. No recurrence of AIHA was observed during the follow-up period. The progression of laboratory markers and clinical interventions during AIHA in case 1 is shown in figure 1.

Bili: indirect bilirubin (mg/dL); Hb: hemoglobin (g/dL); PRED: oral prednisone; Ret: reticulocyte Count (%). Time is represented in days.

### Case 2

Case 2 was a 12-year-old boy with O+ blood type, no parental consanguinity, and no personal or family medical history of AIHA, diagnosed with HbSS disease, and had splenectomy, cholecystectomy, bilateral femoral head necrosis, and chronic hypoxemia. His first RBC transfusions at 8 years of age were ABO, Rh (D, C, c, E, and e), and Kell (K) phenotype-matched units. After the first two transfusions, anti-Fya, anti-Jka, and anti-S alloAbs were identified. During follow-up, the patient received another 16 sporadic transfusions of RBC units, and new alloAbs were detected (anti-Lea and anti-Leb, with thermal amplitude and reactive at 37^o^C). The identification of alloAbs was confirmed after careful review of the indirect antiglobulin test and genotyping.

**Figure 2:**
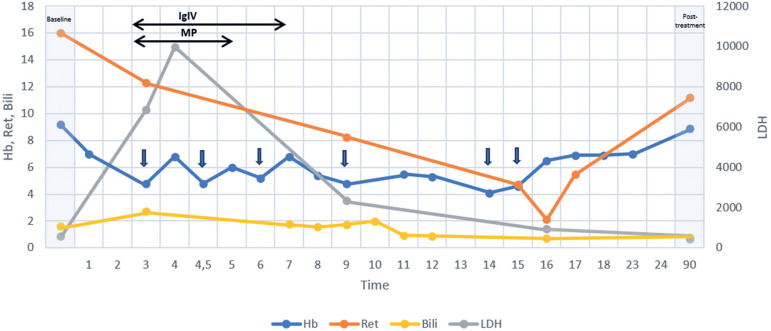
Progression of laboratory markers and clinical interventions during autoimmune hemolytic anemia in sickle cell disease (Case 2)

The patient was admitted to our hospital with sudden worsening of skin pallor, jaundice, weakness, dyspnea, fever, somnolence, tachycardia (heart rate of 158 bpm), tachypnea (49 breaths per minute), hypotension (87/59mmHg), and desaturation (86% oxygen saturation). Cardiac auscultation revealed a gallop rhythm, and the liver was palpable at the costal arch. Blood tests showed worsening of anemia [Hb from 9.1g/dL (baseline) to 4.8g/dL], a drop in reticulocyte absolute number [from 255 × 10^9^/L (baseline) to 80 × 10^9^/L], 65 orthochromatic erythroblasts per 100 WBCs, an increased lactate dehydrogenase level [from 566 U/L (baseline) to >6.000 U/L], and an increased indirect bilirubin level [from 1.6 mg/dL (baseline) to 2.7mg/dL]. The WBC and platelet counts were normal. Direct antiglobulin test revealed undetermined pan-agglutinin (IgG) levels. No new alloAbs were identified. These data, along with the clinical and laboratory signs of hemolysis, led us to confirm the diagnosis of wAIHA. Infections, lymphoproliferative diseases, solid malignancies, and autoimmune diseases (AIDs) were excluded. Due to the severity of the clinical presentation, the patient was treated with corticosteroids and intravenous immunoglobulin (IVIG). He received high-dose pulse methylprednisolone (30mg/kg/day with a maximum dose of 1g/day for 3 days), followed by transition to oral prednisone (2.0mg/kg/day with a maximum dose of 60mg/day for 28 days) and then weaning. Intravenous immunoglobulin was administered at 0.4g/kg/day for 5 consecutive days. Owing to the signs of hemodynamic instability at presentation, the patient was prescribed an RBC transfusion, which was delayed because of the presence of multiple alloAbs and difficulty in finding compatible RBC units. A strategy for the adsorption of anti-Lea and anti-Leb alloAbs was adopted through daily transfusions of fresh frozen plasma units from secretor donors. A decrease in the thermal amplitude of the anti-Lewis antibodies was observed. The patient received a total of seven phenotype-matched (not for Lewis) RBC units during hospitalization, each justified by signs of symptomatic anemia or very low Hb levels (usually <5.0g/dL), with a brief post-transfusion increase in Hb level, further hemolysis, and return to the pre-transfusion Hb level within 48 to 72 hours. The patient developed immunosuppression, with improvement in the signs of hemolysis. Twenty-one days after treatment, the patient's Hb level, reticulocyte count, lactate dehydrogenase level, and indirect bilirubin level were at baseline. The DAT results became negative 2 years later. No recurrence of AIHA was observed during the follow-up period. The progression of laboratory markers and clinical interventions during AIHA in case 2 is shown in figure 2.

Bili: indirect bilirubin (mg/dL); Hb: hemoglobin (g/dL); IgIV: intravenous immunoglobulin; LDH: lactate dehydrogenase (U/L); MP: methylprednisolone pulse; Ret: reticulocyte Count (%). Time is represented in days. Arrows represent days in which the patient received RBC transfusions.

## DISCUSSION

Sickle cell disease is the most prevalent and clinically significant inherited blood disorder worldwide. AIHA is rare in general pediatric patients, with an annual incidence of 0.81/100,000 in patients under 18 years of age.^(5)^ Autoimmune hemolytic anemia in general pediatric patients is uncommon in adolescents (14%), and its prevalence according to sex is controversial.^(6)^ However, the age at the time of autoantibody formation in both patients 1 and 2 was similar to that previously reported in the literature for patients with SCD.^(3)^ Some studies have focused on the coexistence of pediatric SCD and AIHA. However, the prevalence and incidence rates of these conditions remain unknown.

In this report, both patients presented with severe phenotypes of SCD. Recent studies have demonstrated an important relationship between a higher chronic inflammatory status and manifestation of more severe clinical symptoms, suggesting a crucial role of the immune system in SCD pathophysiology. Chronic hemolysis promotes heme release, which induces sterile inflammation via toll-like receptor-4 and inflammasome signaling. This leads to the activation of neutrophils, monocytes, and lymphocytes, as well as an increase in proinflammatory cytokine levels.^(7)^ The neutrophil-to-lymphocyte ratio (NLR) is one of the most common indicators of inflammatory processes in SCD.^(8)^ It has also been described an hyperactivation of the complement system,^(9)^ and polyclonal B cell activation, leading to the production of autoAbs. These autoAbs exacerbate inflammation by forming immune complexes that activate proinflammatory pathways, recruit immune cells, and contribute to tissue damage.^(10)^ Furthermore, repeated deformation of the erythrocyte membrane and abnormal interactions with the endothelium may lead to the exposure of erythrocyte neoantigens that induce IgG autoantibody formation.^(3)^ Autoantibody formation may also be enhanced by the abnormal modification and externalization of autoantigens, owing to an imbalance between neutrophil extracellular trap (NET) formation and active clearance in SCD.^(11)^ A recent cohort study reported a high prevalence of biological autoimmunity in SCD, with 58% of patients having positive serum autoAbs, of whom 9.7% developed clinical AID with an unfavorable evolution for both AID and SCD. One hypothesis is that during AID activity, proinflammatory cytokines stimulate the vascular endothelium and promote vaso-occlusive crisis.^(12)^

Although patient 1 presented with Hashimoto's thyroiditis, the frequency of SCD and thyroiditis was not evaluated. The most frequently reported AIDs associated with SCD are rheumatoid arthritis and systemic lupus erythematosus;^(9)^ however, most data, especially in children, are from case reports,^(13)^ and the coexistence of SCD and AIDs is still likely underdiagnosed.

Approximately 25% of patients with AID tend to develop additional AIDs.^(14)^ This risk may be due to genetic susceptibility that affects both diseases and the alteration of the body's homeostasis by one disease that creates susceptibility to another or some as-yet-undefined shared mechanism.^(15)^ However, there are few reports on the association between Hashimoto's thyroiditis and AIHA.

The benefits of HU in patients with SCD are attributed to elevated fetal Hb levels, as well as total Hb and mean cell volume of red cells, reduced WBC, platelet, and reticulocyte counts, downregulated adhesion molecules, and increased nitric oxide release. Although HU significantly improves most hematological parameters, the levels of inflammatory cytokines (tumor necrosis factor alpha and interleukin-6) and the NLR in patients with SCD do not reach values similar to those in healthy controls.^(8)^ Therefore, despite receiving HU, patient 1's inflammatory state, associated with other conditions, could have been related to the occurrence of AIHA. Although the anemia was not severe, the role of HU in modulating the autoimmune response has not yet been clarified. In addition, despite patient 1 presenting with no alloAbs, HU treatment does not reduce the likelihood of alloimmunization.^(7)^ Patient 2 did not receive HU, which could have contributed to the intense oxidative stress and inflammatory state that may have been related to the severe clinical presentation of AIHA.

Both presented cases had previously undergone splenectomy. Significant autoAb titers have been reported after splenectomy, and recent experimental studies have investigated the role of splenectomy in the autoimmune response. The hypothesis is that the absence of the spleen could be compensated by other peripheral lymphoid organs^(13)^ with overproduction of autoAbs. A recent cohort study associated a history of splenectomy with a higher risk of AID in SCD and raised the possibility that decreased clearance of immune complexes occurs after splenectomy.^(12)^

Recent studies have demonstrated an association of chronic inflammation, immune dysregulation, alloreactivity, and autoimmunity in patients with SCD. Alloimmunization is more frequent with SCD. This is partially attributable to ethnic discrepancies and genetic diversity among donors and SCD receptors, especially in Rh blood group system antigen expression.^(16)^ Patient 2 developed multiple alloAbs, likely favored by a chronic inflammatory state and possible dysfunction of regulatory B and T cells.^(17,18)^ The chronic pro-inflammatory state creates a microenvironment with increased levels of inflammatory cytokines, which favors an altered antigen-presenting context for macrophages and dendritic cells.

Although autoAbs are less recognized as a complication of multi-transfused patients with SCD, some studies have reported transfusion exposure (24-72 RBC units) as a risk factor for the development of autoAbs.^(3,4,19)^ Younger age at first transfusion has been associated with a lower incidence of alloimmunization, possibly because of an immature immune system and some form of acquired immune tolerance to allogeneic RBC antigens.^(20)^ However, the association between the early onset of transfusion and autoAbs has not been completely elucidated.^(21)^

The formation of autoAbs may be associated with previous alloimmunization. AlloAbs bind to the RBC membrane, inducing conformational changes and creating neoantigens.^(3,22)^ The neoantigen stimulate the production of antibodies that cross-react with self-antigens. Although no specific alloAbs have been described to strongly induce autoAbs, a high prevalence of autoAbs has been observed among patients alloimmunized with the E, V, S, and Jk^b^ antigens.^(23)^ Other theories to explain the induction of RBC autoAb development after alloimmunization in patients with SCD include failure to regulate alloantibody-induced lymphoproliferation as well as altered processing and presentation of alloantigens to T cells.^(13)^ The most frequent autoAbs identified in SCD are pan-agglutinating IgG and autoAbs with RH-specificity (mainly to "e" antigen).^(1,3)^ Considering that genes in the RH blood group are highly susceptible to recombination events and that variants may be difficult to distinguish serologically, some of the anti-e antibodies identified in the literature were possibly alloAbs developed by individuals with RHD and RHCE variants with weak or partial antigen expression.^(18)^ To date, the relationship between previous alloimmunization and AIHA severity is unknown. Furthermore, erythrocyte allo-immunization is not necessary for autoantibody formation.

Clinically significant autoimmune hemolysis is a rare but potentially life-threatening complication of autoimmunization. For general pediatric patients, the first-line therapy for wAIHA is corticosteroids at different doses and time courses. In more severe cases, IVIG has been used in addition to steroids as adjunctive therapy.

## CONCLUSION

Few studies have focused on pediatric sickle cell disease and autoimmune hemolytic anemia. The development of autoAbs and autoimmune hemolytic anemia may be related to a chronic proinflammatory state, immune dysregulation of regulatory B and T cells, alloimmunization, transfusion exposure, and splenectomy in patients with sickle cell disease. No guidelines for managing autoimmune hemolytic anemia in patients with sickle cell disease are currently available. The risk and prognostic factors for autoimmune hemolytic anemia associated with sickle cell disease are potential topics of future research. The description of these rare presentations and their management and evolution may contribute to further studies and improvements in patient care.
